# Factors associated to referral of tuberculosis suspects by private practitioners to community health centres in Bali Province, Indonesia

**DOI:** 10.1186/1472-6963-13-445

**Published:** 2013-10-28

**Authors:** I Wayan Gede Artawan Eka Putra, Ni Wayan Arya Utami, I Ketut Suarjana, I Made Kerta Duana, Cok Istri Darma Astiti, IW Putra, Ari Probandari, Edine W Tiemersma, Chatarina Umbul Wahyuni

**Affiliations:** 1 School of Public Health, Faculty of Medicine, Udayana University, Bali, Indonesia; 2 NTP of Bali Province Health Office, Bali, Indonesia; 3 NTP of Tabanan District, Health Office, Bali, Indonesia; 4 Department of Public Health, Faculty of Medicine, Universitas Sebelas Maret, Surakarta, Indonesia; 5 KNCV Tuberculosis Foundation, The Hague, The Netherlands; 6 Faculty of Public Health, Airlangga University, Surabaya, Indonesia

**Keywords:** Tuberculosis, Private practitioners, Operational research, Indonesia

## Abstract

**Background:**

The contrast between the low proportion of tuberculosis (TB) suspects referred from private practitioners in Bali province and the high volume of TB suspects seeking care at private practices suggests problems with TB suspect referral from private practitioners to the public health sector. We aimed to identify key factors associated with the referral of TB suspects by private practitioners.

**Methods:**

We conducted a case-control study conducted in Bali province, Indonesia. The cases were private practitioners who had referred at least one TB suspect to a community health centre between 1 January 2007 and the start of data collection, while the controls were private practitioners who had not referred a single TB suspect in the same time.

**Results:**

The following factors were independently associated with referral of TB suspects by private practitioners: having received information about the directly observed treatment short-course (DOTS) strategy (OR 2.0; 95% CI 1.1 – 3.8), ever having been visited by a district TB program officer (OR 2.1; 95% CI 1.0 – 4.5), availability of TB suspect referral forms in the practice (OR 2.8; 95% CI 1.5-5.2), and less than 5 km distance between the private practice and the laboratory for smear examination (OR 2.2; 95% CI 1.2-4.0).

**Conclusions:**

Education and exposure of private practitioners to the TB program improves referral of TB suspects from private practitioners to the national TB program. We recommend that the TB program provides all private practitioners with information about the DOTS strategy and TB suspect referral forms, and organizes regular visits to private practitioners.

## Background

Indonesia has a high burden of tuberculosis (TB) with approximately 450,000 new patients in 2010 [1]. Multidrug resistant (MDR) TB, which is more difficult to treat, may emerge if TB is not appropriately treated and has been associated with the presence of an important private sector [2]. Private practitioners often do not follow the directly observed therapy, short course (DOTS) strategy [3]. Several studies showed that poor adherence to the DOTS strategy leads to poor treatment outcomes [4]. To increase the coverage of DOTS treatment, the involvement of private practitioners in a TB control program is essential [5,6].

Although the absolute contribution to TB case finding and treatment is not exactly known for most countries, some studies from different Asian countries suggest that private practitioners may play an important role in the diagnosis and treatment of TB [6,7]. In Indonesia, a national survey on health seeking behaviour among TB suspects in 2004 showed that 54% first sought treatment with private practitioners [8]. A study involving telephone interviews of 25% of the private practitioners in Jogjakarta province showed that most private practitioners (63%) reported to have seen TB suspects in their private practice. Almost one-third of the interviewed private practitioners never referred a TB suspect to the national TB control program (NTP) for diagnosis and treatment and most of these private practitioners did not offer DOTS treatment [9]. A study in Bali reported three main problems associated with TB care within the private sector: poor TB treatment adherence among patients, limited capacity of public services and poor public-private integration [10].

Since 2004, a partnership program in Bali has encouraged 537 private practitioners (23 specialists, 161 medical doctors, 189 midwives, and 164 nurses) to participate in the TB control program. However, only 2.8% of TB suspects identified by the TB control program had been referred to a community health centre by a private practitioner [11]. This finding contrasts with the high volume of TB suspects seeking care at private practices as found in the above-mentioned studies and suggests problems with TB suspect referral from private practitioners to the public health sector. We aimed to identify factors associated to referral of TB suspects by private practitioners in Bali, Indonesia.

## Methods

### Design

We performed a case-control study. A case was defined as a private practitioner who had referred a TB suspect between 1 January 2007 and the start of data collection. A control was defined as a private practitioner who had not referred any TB suspect to the public sector over the same time period.

### Study settings

The study was conducted in Tabanan and Karangasem districts in Bali province, Indonesia. Tabanan is located in West Bali and Karangasem in East Bali. These districts were selected because they are representative for whole of Bali in many respects. While many private practitioners serve in both areas, a relatively low proportion of all TB suspects registered in community health centres in Tabanan district had been referred by a private practitioner (1.1%) in 2007, whereas this proportion was relatively high for Karangasem (14.1%). Tabanan and Karangasem are rural districts. In 2007, Tabanan and Karangasem were inhabited by 407,162 (485 persons/km^2^) and 382,939 persons (456 persons/km^2^), respectively. In 2007, there were 61 physicians and 324 midwives/nurses (paramedical staff examining and treating patients especially in remote areas) in Tabanan and 47 physicians and 247 midwives/nurses in Karangasem [12,13].

### Study population, sample size and sampling design

The study population consisted of private practitioners in Bali Province including pulmonologist, internists, general practitioners, midwives and nurses. We used Kelsey’s formula [14] for sample size calculations, and assumed that the proportion of private practitioners exposed to information about the DOTS program was 25% among cases (P_1_) and 10% among controls (P_0_). We calculated that a minimum sample size of 92 in each group was needed, taking a ratio of cases to controls of 1:1, a significance level of 5% and a study power of 80%. We aimed to include 100 persons per group to account for losses due to partial participation.

The study used multistage cluster randomized sampling. First, Tabanan and Karangasem district were selected purposively from nine districts in Bali, because the two districts represented the extremes in the contribution of private practitioners to the referral of TB suspects to community health centres (see above). We randomly selected 10 community health centres in each district. Then, we randomly selected 10 private practitioners in the coverage area of each of the selected community health centres from a list that contained all private practitioners in the two districts by type of practitioner.

### Data collection and analysis

Data were collected by interviewers using pre-structured questionnaires that had been piloted among 15 private practitioners during a small pilot study in another district (Gianyar). We collected information about the characteristics of the private practitioners (gender, age, occupation, and qualification), work experience, opening hours per day, availability of TB suspect referral forms in the private practice, and distance of the participant’s private clinic to a sputum smear laboratory. Also, we asked whether the respondent had ever received information about DOTS, whether or not the participant could remember to have been visited at least once by a TB officer (the officer who is responsible for the conduct of the TB program in districts). We assessed the respondent’s knowledge about the DOTS strategy by asking 11 multiple-choice questions. The questions covered three basic competencies that medical doctors should have in relation to TB case management, i.e. TB suspect identification, diagnosis and treatment. The 11 questions covered signs and symptoms of adult and childhood TB, laboratory diagnosis, diagnosis of TB in children, number of sputum samples to be analysed for diagnosis of TB, type of sputum examination for diagnosis, timing of the start of TB treatment, anti TB drugs, cost of treatment, duration of treatment and the importance of direct observation. Good knowledge was defined as having answered more than 5 of these 11 questions correctly, while having less than five correct answers was categorized as poor knowledge.

We trained four interviewers to perform the data collection. The district TB officers approached the selected private practitioners. If they agreed to participate, an appointment for interview was made. The private practitioners were interviewed in their office by the study team.

Of 200 private practitioners who had been invited to participate to the study, all agreed to be interviewed. However, only 181 (90.5%; 94.0% of the cases and 87.0% of the controls) were present at the appointed date and time of the interview. The private practitioners who could not be interviewed were more often specialist (pulmonologist and internist) than those who were interviewed. A private practitioner who was not available at the time of interview was replaced by the private practitioner appearing above the selected private practitioner on the list. If this private practitioner was not available at the time of the interview, the practitioner below the initially selected practitioner was chosen instead, so that the total study population included 100 cases and 100 controls.

We used Chi square tests to identify univariate associations between private practitioner characteristics and TB suspect referral. Backward logistic regression models included all variables with a p value < 0.25 in the univariate analysis (Chi square test).

The ethical clearance was obtained from the Ethics Committee of the Faculty of Medicine, Udayana University and the permission to implement the study was obtained from the National Unity and Public Protection Body (Kesbanglinmas), Bali Province.

## Results

Of a total of 200 private practitioners, 100 practitioners who had referred at least one TB suspect to the TB control program (cases) between 1 January 2007 and the start of data collection and 100 practitioners who had not done this (controls) were included in the study. The general characteristics of the cases and controls are given in the table. In comparison to controls, cases were more often of male (49% vs 33%). Also, the proportion of physicians was higher among cases than among controls (24% vs 16%), but this difference was not statistically significant. There were no differences with respect to the other characteristics listed in the table.

Most respondents (96%) lacked knowledge about the diagnosis of TB in children and only part of them (49%) was able to correctly indicate when TB therapy should be started. Almost all respondents correctly answered the other knowledge questions, such as the need of appointing a treatment observer, the duration of TB treatment and the anti-TB drugs that constitute a standard TB regimen. There was no significant difference in overall knowledge between private practitioners who referred TB suspects and those who did not refer TB suspects to the health centre (OR 1.6; 95% confidence interval (CI) 0.6-4.4).

Among the practitioners who reported to have requested sputum smear examination from a laboratory at least once in the past year (N = 100), 28% reported that they had never received the results of the examination. Private practitioners who had referred TB suspects to public health centres were more likely to have received information about the DOTS strategy (OR 2.2; 95% CI 1.1-3.5), been visited at least once by a TB officer (OR 2.9; 95% CI 1.5-5.8), have TB suspect referral forms available (OR 2.9; 95% CI 1.6-5.2) and have their clinic at a distance of less than 5 km from a sputum smear laboratory (OR 2.6; 95% CI 1.5-4.5). The multivariate analysis showed that all these factors were independently associated with referring TB suspects to a public health facility (Table 1).

**Table 1:** Factors associated to referral of TB suspects to community health centres by private practitioners in Bali province, Indonesia

**Characteristics**	**Cases N = 100**	**Controls N = 100**	**Univariate analysis**		**Multivariate analysis‡**	
			OR*	**95% CI†**	OR*	**95% CI†**
**Characteristics of the respondents**						
Gender, % male	49	33	1.9	1.1-3.5	--	-
Age, % < 40 years	48	51	0.9	0.5-1.5	--	-
Occupation, % government employee	98	98	1.0	0.1-7.2	--	-
Medical doctor, %	24	16	1.7	0.8-3.4	--	-
Education, % bachelor or higher	26	17	1.7	0.9-3.4	--	-
Work experience, % ≤ 5 years	33	36	0.9	0.5-1.6	--	-
**Knowledge about DOTS: % of private practitioners with correct answers**					--	
Typical signs and symptoms classifying a patient as a TB suspect	75	72	1.2	0.6-2.2	--	-
Typical signs and symptoms classifying a child as a TB suspect	44	55	0.6	0.4-1.1	--	-
Laboratory diagnostics for TB suspects	95	87	2.8	0.98-8.3	--	-
Diagnostics for children suspected of TB	3	5	0.6	0.1-2.5	--	-
Frequency and moment of examination of sputum sample	79	73	1.4	0.7-2.7	--	-
Type of sputum examination for diagnosis	61	63	0.9	0.5-1.6	--	-
Timing of the start of TB treatment	49	59	0.7	0.4-1.2	--	-
Drugs used in TB treatment regimen	95	93	1.4	0.4-4.7	--	-
Costs of TB treatment	100	95	-	-	--	-
Duration of treatment	99	93	7.5	0.9-61.7	--	-
Direct observation of TB patient swallowing medicine needed	100	95	-	-	--	-
**Overall knowledge, % good**	84	76	1.6	0.6-4.4	--	-
**Support received from TB control program**						
At least once visited by TB officer, %	34	15	2.9	1.5-5.8	2.1	1.03-4.5
Received information about DOTS strategy, %	76	59	2.2	1.2-4.0	2.0	1.1-3.8
**Characteristics of the clinic**						
Clinic opening hours per day, % ≤ 2 hours	14	10	1.5	0.6-3.5	--	-
TB suspect referral forms available, %	54	29	2.9	1.6-5.2	2.8	1.5-5.2
Distance between clinic and nearest laboratory, % < 5 km	64	41	2.6	1.5-4.5	2.2	1.2-4.0

*OR = odds ratio; † CI 95% = 95% confidence interval; ‡ odds ratios are adjusted for all other variables that are displayed with an odds ratio in this table.

## Discussion

The results of this study reveal that the provision of information about the DOTS strategy and referral of TB suspects are important to improve the referral of TB suspects by private practitioners to public health centres. Private practitioners who had referred TB suspects were more likely to have been visited by a TB officer, have received information about the DOTS strategy, and to have referral forms available than the practitioners who had not referred a single TB suspect. A TB officer, when visiting a private practitioner, is expected to remind practitioners about their role in finding TB suspects and about the importance of sending TB suspects to public health facilities for microscopic evaluation.

A study in Myanmar concludes that the success factors of the private practitioners’ contribution to TB control were training and supervision by the public sector and provision of drugs and consumables free of charge by the NTP [15]. A study about the process of collaboration between NTP and hospitals in Yogyakarta, Indonesia, reveals that partnership can be established if it begins with an intensive process of interaction including intermediary actors (i.e., persons approaching the private hospital and advocating the partnership program) for approaching the NTP and the hospital [16]. Other studies from different countries and on different continents show that the success of public-private mix projects largely depends on the level of interaction between private and public health care providers [17,18].

Our study also indicated important enablers of referral: the availability of official TB suspect referral forms and of a smear examination laboratory within the vicinity of the private clinic. Private practitioner’s patients who had been referred to a public facility were more likely to have visited a microbiology laboratory located within 5 km distance of the private clinic. The balance between the benefit of receiving a laboratory examination and the time and costs needed to get to the laboratory is more likely to be rated positive by TB suspects if they have a laboratory for sputum smear nearby. Private practitioners having referral forms available were twice more likely to refer suspects than those who did not have such forms. Referral slips were successfully introduced in private practices earlier [19], but to our knowledge, this is the first study indicating that the presence of referral forms alone is an important enabler for referral of TB suspects from private practitioners to the public sector.

An important factor that may discourage private practitioners to refer TB suspects is the lack of feedback from the community health centre about the sputum examination result of the TB suspect that they referred. Almost one third of private practitioners who had referred TB suspect(s) to a sputum smear laboratory declared that they had never received the sputum smear result of these suspects. Private practitioners not receiving any feedback on the test results of TB suspects can be expected to get discouraged in referring suspect because they feel not appreciated for their participation and they also may get the impression of losing patients because these do not go back to private practitioners. Indeed, when private practitioners were asked about their wishes with respect to public-private collaboration, most of them indicated that they desired to receive adequate and timely feedback of the public health facilities (data not shown).

Our study had some limitations. First, we included only part of the private practitioners from two districts in Bali province. However, since these two districts represent the extremes in TB suspect referral and since private practitioners were randomly selected, we are convinced that this study gives a good representation of private practitioner’s characteristics related to referral practices in Bali. Second, we selected private practitioners as controls if they had not referred a TB suspect from January 2007 until the start of data collection, and as cases if they had done this. The probability of referring a TB suspect to the public health sector may not only depend on the private practitioner’s characteristics, but also on the clinic’s patient load. Practitioners who see more potential TB suspects per day may have more experience in identifying symptoms of pulmonary TB and are more likely to refer pulmonary TB suspects than private practitioners who rarely see patients with respiratory symptoms. Out of efficiency considerations, the larger practices may have received more attention from the TB officer in the past than smaller clinics. However, assuming that the opening hours per day can be regarded as a proxy for the clinic’s patient load, we found no indications for such an effect, as the proportion of private practices with opening hours of ≥ 2 hours per day did not differ between the cases and controls (86% at case and 90% at control group, p > 0.05).

This study provides important information to the TB control program about what factors enable the referral of TB suspects by private practitioners to public health care providers. The major benefit of the study is indirect, however. The provincial health office was closely engaged in this study from its start. The results from this study have generated a sense of urgency to engaging private practitioners in TB case finding in Bali. As an indirect outcome of this study, an intervention is being prepared to improve the engagement of private practitioners in Bali province. The intervention is an innovative program of awarding participation credit points (PCP) to private practitioners that refer TB suspects to public health facilities in order to improve their collaboration to the existing public-private mix projects. The effect of this intervention on TB case finding will be closely monitored, and, if positive, the intervention will be expanded to other Indonesian provinces.

## Conclusions

To improve private practitioners’ involvement in TB case finding, district TB officers should invest in visiting private clinics to educate private practitioners about the DOTS program, and to provide referral forms of TB suspect. Also, bringing laboratory services closer to the clinic, e.g. by implementing a sample transportation system, may enable TB case finding in Bali. All private practitioners should receive continuous supportive supervision and should be provided with high quality information about the DOTS program and referral forms for TB suspects. Most importantly, this operational study has provoked new interventions by the provincial TB office to increase participation of private practitioners in the diagnosis of TB in Bali province.

## Competing interests

The authors declare that we have no competing interests.

## Authors’ contributions

IWGAEP designed the study, developed the data collection tools, conducted the interviews, data analysis and drafted the manuscript. UNWA contributed to the design of the study, developed the methodology, and helped designing the data collection tools. IKS contributed to designing the study, development of the methodology and collected the data. IWP provided background information about the TB program in Bali province and helped in designing the data collection tools. CIDA contributed to development of the data collection tools, provided TB related epidemiological information and helped with data collection. IMKD contributed to data collection and drafting of the manuscript. AP, EWT and CUW critically reviewed all aspects of the study and assisted in writing the manuscript. All of the authors have approved the final manuscript.

## Pre-publication history

The pre-publication history for this paper can be accessed here:

http://www.biomedcentral.com/1472-6963/13/445/prepub
